# ASSOCIATION OF WELL-BEING WITH ANXIETY, DEPRESSION, AND FUNCTIONAL IMPAIRMENT FOLLOWING REHABILITATION SERVICES

**DOI:** 10.1093/geroni/igz038.1009

**Published:** 2019-11-08

**Authors:** Adam Simning, Christopher L Seplaki

**Affiliations:** University of Rochester, Rochester, New York, United States

## Abstract

Millions of older adults receive rehabilitation services yearly that aim to restore, sustain, or limit decline in functioning. Older adults who receive rehabilitation comprise a vulnerable population that is unfortunately at elevated risk for anxiety, depression, and functional impairment. We hypothesize that lower levels of wellbeing prior to rehabilitation services are associated with a greater risk of having clinically significant anxiety or depressive symptoms, or worsening impairments in self-care or household activities, following rehabilitation. This study uses data from 2015 and 2016 waves of the National Health and Aging Trends Study, and includes 853 participants with information on rehabilitation services, wellbeing, anxiety and depression, and functional impairment, as well as demographic characteristics, socioeconomic status, and health variables. In a series of multivariable logistic analyses with wellbeing serving as our primary independent variable, older adults in the lowest quartile of wellbeing (compared to those in the highest quartile of wellbeing) had greater odds for having anxiety symptoms (OR=3.04; 95% CI: 1.24-7.46), depressive symptoms (OR=6.54; 95% CI: 2.80-15.25), and worsening impairment in self-care (OR=2.15; 95% CI: 1.09-4.23), but not in household activities (OR=1.49; 95% CI: 0.67-3.32). This study’s findings suggest that older adults with low levels of wellbeing at baseline may be more susceptible for having mental illness and functional impairment at follow-up. Conversely, the findings suggest that perhaps those with high levels of wellbeing may be able to experience significant health events with fewer residual consequences. The mechanism by which wellbeing may affect these outcomes is unclear and warrants further investigation.

